# Molecular characterization of gastric adenocarcinoma diagnosed in patients previously treated for Hodgkin lymphoma or testicular cancer

**DOI:** 10.1371/journal.pone.0270591

**Published:** 2022-07-25

**Authors:** Lisanne S. Rigter, Petur Snaebjornsson, Efraim H. Rosenberg, Estelle Altena, Nicole C. T. van Grieken, Berthe M. P. Aleman, Jan M. Kerst, Lindsay Morton, Michael Schaapveld, Gerrit A. Meijer, Flora E. van Leeuwen, Monique E. van Leerdam, PALGA group

**Affiliations:** 1 Department of Gastroenterology, Netherlands Cancer Institute, Amsterdam, The Netherlands; 2 Department of Pathology, Netherlands Cancer Institute, Amsterdam, The Netherlands; 3 Department of Pathology, VU University Medical Center, Amsterdam, The Netherlands; 4 Department of Radiation Oncology, Netherlands Cancer Institute, Amsterdam, The Netherlands; 5 Department of Medical Oncology, Netherlands Cancer Institute, Amsterdam, The Netherlands; 6 Division of Cancer Epidemiology and Genetics, National Cancer Institute, Bethesda, Maryland, United States of America; 7 Department of Epidemiology, Netherlands Cancer Institute, Amsterdam, The Netherlands; Chinese Academy of Sciences, CHINA

## Abstract

**Introduction:**

The risk of developing gastric cancer is increased in patients treated with radiotherapy for Hodgkin lymphoma (HL) or testicular cancer (TC). This study aims to assess if gastric adenocarcinoma after treatment for HL/TC (t-GC) is molecularly different from gastric adenocarcinoma in the general population.

**Methods:**

Patients were diagnosed with t-GC ≥5 years after treatment for HL/TC. Four molecular subtypes were identified using immunohistochemical and molecular analyses: Epstein-Barr virus (EBV), mismatch repair (MMR) deficiency or microsatellite instability (MSI), aberrant p53 staining as surrogate for chromosomal instability (sCIN), and a surrogate for genomic stability (sGS) without these aberrations. Results were compared with gastric cancer in the general population (p-GC) described in literature.

**Results:**

Molecular subtyping of 90 t-GCs resulted in 3% EBV, 8% MSI, 36% sCIN and 53% sGS. 3/6 of MSI t-GCs had *MLH1* promoter methylation and 2/6 were explained by double somatic mutations in MMR genes. T-GCs were more frequently sGS than p-GCs (53% vs. 38%, *p =* 0.04). T-GC was more frequently sGS in HL/TC patients diagnosed before 1990, than after 1990 (63% vs. 38%, *p =* 0.03). T-GCs located in the antrum, an area that receives high irradiation doses, were more frequently sGS (61% vs. 28% in p-GCs, *p =* 0.02).

**Conclusion:**

Our results demonstrate that t-GCs are more frequently of the sGS subtype than p-GCs. An association of t-GC of the sGS subtype with prior anticancer treatment is suggested by the high frequency in HL/TC patients who were treated before 1990, a time period in which HL/TC treatments were more extensive.

## Introduction

The risk of developing gastric cancer is increased in survivors of Hodgkin lymphoma and testicular cancer [1–16]. Compared with the general population, 2- to 13-fold increased risks have been reported, which persist up to 40 years after primary cancer treatment [5, 11]. In survivors of both Hodgkin lymphoma and testicular cancer, prior treatment with abdominal radiotherapy is strongly associated with the increased gastric cancer risk [2, 3, 9–11]. In Hodgkin lymphoma survivors, the alkylating agents procarbazine and possibly dacarbazine are also associated with the increased risk of gastric cancer [2].

Overall survival of gastric cancer has been reported to be worse in Hodgkin lymphoma survivors compared with first primary cancer patients, which could result from patient-related factors (e.g. comorbidity or reduced treatment options due to prior cancer treatment), but may also reflect a difference in tumor biology [17].

Although several mouse models described alkylating agents, and irradiation to a lesser extent, as gastric carcinogens, the role of these treatments in the pathogenesis of gastric adenocarcinoma is unknown [18–20]. Our group previously studied therapy-related colorectal cancer in Hodgkin lymphoma survivors and demonstrated a high frequency of microsatellite instability (MSI) compared with the general population. This high MSI frequency in therapy-related colorectal cancer was not caused by *MLH1* promoter methylation, which is common in sporadic colorectal cancer, but was the consequence of somatic mismatch repair (MMR) gene mutations [21]. In a small study on therapy-related esophageal cancer, however, MSI frequency was low [22].

In the general population, approximately 7–23% of gastric adenocarcinomas are MSI [23–27]. These adenocarcinomas are considered a separate molecular subtype in the classifications of The Cancer Genome Atlas Research Network (TCGA) and the Asian Cancer Research Group (ACRG) [23, 24]. Microsatellite stable gastric adenocarcinomas are further subdivided into Epstein-Barr virus (EBV)-positive (9%), chromosomally unstable (50%) and genomically stable (20%) subtypes according to TCGA [23]. The distribution of these molecular subtypes is influenced by multiple factors, including gastric cancer location, age at diagnosis and population [23, 28, 29]. As an alternative for the extensive molecular characterization used by both TCGA and ACRG, surrogate molecular classifications using immunohistochemistry have been developed [25–27].

The molecular subtype distribution of gastric adenocarcinoma after treatment (t-GC) for Hodgkin lymphoma or testicular cancer is unknown. In both survivors of Hodgkin lymphoma and testicular cancer, the increased risk is associated with cancer treatment, which may result in a different pathogenesis of second primary cancers in both groups compared to sporadic gastric cancer. We hypothesize that t-GCs may have a high frequency of EBV, as 30% of Hodgkin lymphomas are associated with EBV. A second *a priori* hypothesis is that t-GCs may have a high frequency of chromosomal instability due to the effects of radiotherapy. Finally, a high frequency of MSI caused by somatic MMR gene mutations may be present, as was reported in therapy-related colorectal cancer [21].

In this study, we evaluate tumor characteristics and molecular subtypes of t-GC, using a surrogate classification method that was previously reported, in order to give insight into the pattern of t-GC development [27].

## Methods

### Patients and tissue samples

This cohort study included patients who were diagnosed with gastric cancer at least five years after the diagnosis of Hodgkin lymphoma or testicular cancer. These patients were identified in two ways.

In the first approach, we identified patients through two Dutch multicenter cohorts of 5-year survivors, one of Hodgkin lymphoma survivors (N = 3905; treatment period 1965–2000) and one of testicular cancer survivors (N = 1029; treatment period 1965–1995) [5, 30]. Information on second cancers, including dates of diagnosis, morphologic features, topographic features and treatment, was collected by a review of medical records, by responses to questionnaires sent to general practitioners, and by record linkage with the Netherlands Cancer Registry since 1989, when the Netherlands Cancer Registry reached nationwide coverage, as described previously [5, 30–32]. The formalin-fixed paraffin-embedded (FFPE) t-GC and normal gastric tissue, and pathology reports of the t-GC patients were retrieved via the nationwide network and registry of histopathology and cytopathology in the Netherlands (PALGA) [33].

The second approach consisted of the identification of additional t-GC patients through the PALGA registry. This registry was searched for patients with a combination of firstly a Hodgkin lymphoma or testicular cancer diagnosis (seminoma or non-seminoma) and secondly a gastric adenocarcinoma diagnosis. Additionally, FFPE tissue and pathology reports of t-GCs and normal gastric tissue were requested. For treatment information on Hodgkin lymphoma and testicular cancer, local hospitals were contacted through PALGA to provide these data anonymously. Unfortunately, treatment data retrieval through this last method was limited due to low hospital participation rates and inaccessible old patient records.

This study was approved by the institutional research board of the Netherlands Cancer Institute (study number CFMPB307). The regulations of the ‘Code for Proper Secondary Use of Human Tissue in The Netherlands’, Dutch Federation of Biomedical Scientific Societies, the Netherlands were applied in collection, storage and use of patient-derived tissue and data. Thus, all data and tumor tissues were processed anonymously.

### Histopathology

H&E stained slides were used for confirmation of the diagnosis of an adenocarcinoma in the stomach or gastroesophageal junction. As the location of adenocarcinoma biopsies of the distal esophagus, gastroesophageal junction, or cardia was poorly defined in pathology reports, and classification systems have changed over time, these tumors were defined as gastroesophageal junction tumors [34]. The reassessment of the diagnosis occurred by standard protocol and included histopathological typing according to Lauren and World Health Organization (WHO) classifications [29, 35], tumor location and tumor-node-metastasis (TNM) stage. For the assessment of distant metastases (M stage), clinical information in pathology reports and tissue samples of metastases were used when available.

### Immunohistochemistry

Tissue microarrays (TMAs) were made if resection specimens were available and used for immunohistochemistry (IHC). When only biopsy specimens were available, whole slides were used. EBV-encoded small RNAs (EBER) in situ hybridization (ISH, INFORM EBER Probe (Roche)) staining was performed on a BenchMark Ultra autostainer (Ventana). For the evaluation of MMR proteins, IHC was performed according to standard protocols for Ventana immunostainer (MLH1 (M1, 6472966001 Roche (Ventana)), MSH2 (G219-1129, 5269270001, Roche (Ventana)), MSH6 (EP49, AC-0047, Epitomics), PMS2 (EP51, M3647, Dako)). IHC of p53 was performed using the DO-7 antibody (Dako). Diffuse, strong nuclear staining (≥70%) was interpreted as aberrant. Human epidermal receptor 2 (HER2) was evaluated by clone 4B5, Roche (Ventana). IHC HER2 scores of 2+ were further analyzed through ISH.

### Molecular analyses

Tumor DNA and normal tissue DNA were isolated using a Qiagen extraction kit. The normal tissue used for DNA isolation included normal gastric mucosa, or adjacent normal tissues of, for example, esophagus or small intestine. DNA concentrations were measured using the Qubit 2.0 Fluorometer with the Qubit dsDNA Assay Kit. MSI was assessed by a pentaplex PCR-based assay using fluorescent labelled primers of five mononucleotide repeat targets (BAT25, BAT26, NR24, NR21, NR27), followed by fragment analysis. In case of instability in two markers or more, the tumor was defined as MSI and further analysis of promoter methylation of MMR genes was performed by multiplex ligation-dependent probe amplification (MLPA, ME011-B2 kit; MRC Holland, Amsterdam, the Netherlands). This included a total of 19 probes located at the promoter region of six different MMR genes (*MLH1*,

*MSH2*, *MSH6*, *PMS2*, *MSH3*, *MLH3*). The presence of promoter methylation was defined as positivity of 33% of probes per gene with a 0.2 cut-off at probe level.

In MSI cases without MMR gene promoter methylation, MMR genes were additionally screened for mutations with the Ion Torrent Personal Genome Machine (Life Technologies, Carlsbad, CA, USA) and pathogenicity was predicted as previously described [21]. In addition, for detection of large genomic aberrations or loss of heterozygosity (LOH) of chromosomes 2, 3 and 7, primer panels for analysis of polymorphic single nucleotide polymorphisms (SNPs) were added.

### Molecular subtypes

All adenocarcinomas were divided into four molecular subtypes, according to the method used in the general population cohort that was used for comparison [27]. This general population cohort consisted of 104 gastric adenocarcinoma patients who were diagnosed at an average age of 67 years (range 34–95 years) in the United States.

Firstly, EBER positive tumors were classified as the EBV subtype. Secondly, the MSI subtype included tumors that displayed aberrant MMR staining and/or MSI. Thirdly, strong nuclear staining of p53 was used as a surrogate for the chromosomal instability (sCIN) subtype, as they frequently have *TP53* mutations [23]. The remaining cases had none of the aberrations (EBER negative, microsatellite stable, no overexpression of p53). This profile represented the fourth subtype, which is surrogate for genomic stability (sGS).

### Statistical analyses

IBM SPSS V.22.0 database software was used to store and analyze data on patient and tumor characteristics. Frequencies of tumor characteristics were compared within subtypes using χ^2^ tests or Fisher’s exact tests for binary or categorical data and Kruskal Wallis tests for continuous data. In addition, these tests were performed for comparison with data from a previously published gastric cancer cohort from the general population that used similar IHC methods to define molecular subtypes [27]. The significance level was defined as two-sided *p*<0.05. No adjustments were made for multiple comparisons.

## Results

### Characteristics of patients who survived Hodgkin lymphoma or testicular cancer and developed gastric cancer

In total 196 t-GC patients were identified. (S1 Fig) FFPE adenocarcinoma tissue was obtained from 90 (46%) t-GCs, diagnosed in 66 Hodgkin lymphoma survivors and 24 testicular cancer survivors. (Table 1) Patients were diagnosed with Hodgkin lymphoma or testicular cancer between 1967 and 2009, at a median age of 39 years (interquartile range (IQR) 27–50 years). Treatment data were available for assessment of treatment category in 43/66 (65%) of Hodgkin lymphoma survivors and 14/24 (58%) of testicular cancer survivors. In 48/57 patients with available treatment data, the treatment consisted of abdominal radiotherapy and/or the alkylating agents procarbazine or dacarbazine.

**Table 1:** Hodgkin lymphoma and testicular cancer characteristics of patients who developed gastric cancer.

Primary cancer characteristics	Total N = 90		Hodgkin lymphoma N = 66		Testicular cancer N = 24	
	median	(IQR)	median	(IQR)	median	(IQR)
Age at primary diagnosis	39	(27–50)	39	(27–52)	44	(29–50)
Latency primary cancer to t-GC in years	16	(11–22)	16	(11–22)	13	(9–22)
	***n***	**(%)**	***n***	**(%)**	***n***	**(%)**
Year of diagnosis						
1967–1979	19	(21)	13	(20)	6	(25)
1980–1989	37	(41)	26	(39)	11	(46)
1990–1999	23	(26)	19	(29)	4	(17)
2000–2009	11	(12)	8	(12)	3	(13)
Abdominal RT						
No	19	(21)	15	(23)	4	(17)
Yes	37	(41)	27	(41)	10	(42)
Unknown	56	(38)	24	(36)	10	(42)
Alkylating agents						
No	30	(33)	16	(24)	14	(58)
procarbazine/dacarbazine	19	(21)	19	(29)	0	(0)
Procarbazine	4	(4)	4	(6)	0	(0)
Dacarbazine	37	(41)	27	(41)	10	(42)
Unknown						
Primary treatment category						
Abdominal RT—/ AA -	9	(10)	5	(8)	4	(17)
Abdominal RT + / AA -	25	(28)	15	(23)	10	(42)
Abdominal RT—/ AA +	11	(12)	11	(17)	0	(0)
Abdominal RT + / AA +	12	(13)	12	(18)	0	(0)
Unknown	33	(37)	23	(35)	10	(42)

***Abbreviations***: t-GC, gastric cancer after treatment for Hodgkin lymphoma or testicular cancer; IQR, interquartile range; RT, radiotherapy; AA, alkylating agents (procarbazine/dacarbazine).

**Fig 1:**
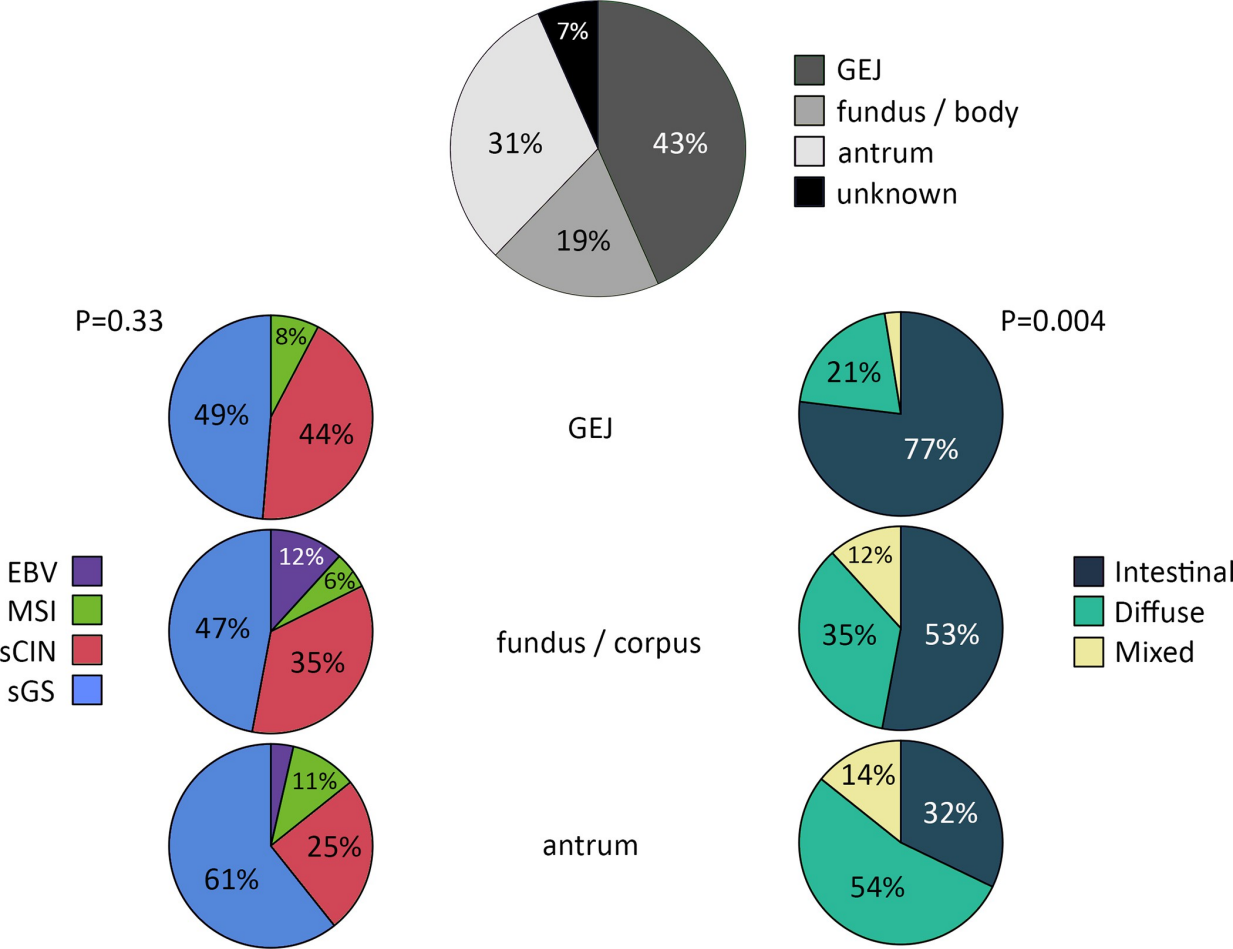
Distribution of molecular subtypes and Lauren classification across locations of gastric cancer after treatment for Hodgkin lymphoma or testicular cancer. ***Abbreviations***: GEJ, gastroesophageal junction; EBV, Epstein-Barr virus; MSI, microsatellite instability; CIN, chromosomal instability; GS, genomic stability.

Patients were diagnosed with t-GC between 1982 and 2015, at a median age of 58 years (IQR 48–69, Table 2). The majority of t-GCs was located at the gastroesophageal junction (43%) and in the antrum (including pylorus, 31%), and 19% of tumors were located in the fundus and corpus of the stomach (Fig 1). TNM stage was evaluable in 57/90 (63%) t-GCs, of which 25/57 (44%) were stage I/II and 32/57 (56%) stage III/IV. Lauren classification resulted in 51 (57%) intestinal type t-GCs, 32 (36%) diffuse type t-GCs and 7 (8%) mixed type t-GCs. The distribution of histological types according to Lauren differed significantly depending on the tumor location; 77% of gastroesophageal junction tumors were of the intestinal type while this type comprised 53% of tumors in the fundus and corpus, and 32% of tumors in the antrum (*p =* 0.004, Fig 1).

**Table 2:** Molecular subtypes of gastric cancer after treatment for Hodgkin lymphoma or testicular cancer.

t-GC characteristic	Total cohort		EBV	MSI	sCIN	sGS
	*n* (%)		*n* (%)	*n* (%)	*n* (%)	*n* (%)
t-GC subtype	90	100	3 (3)	7 (8)	32 (36)	48 (53)
Gender						
Male	70	78	3 (4)	7 (10)	27 (39)	33 (47)
Female	20	22	0	0	5 (25)	15 (75)
Age at t-GC diagnosis						
Median (IQR)	58	(48–69)	43 (42–54)	71 (55–75)	65 (57–77)	52 (42–62)
Year of t-GC diagnosis						
1982–1989	3	3	1 (33)	0	1 (33)	1 (33)
1990–1999	19	21	1 (5)	0	5 (26)	13 (68)
2000–2009	43	48	1 (2)	4 (9)	15 (35)	23 (54)
2010–2015	25	28	0	3 (12)	11 (44)	11 (44)
TNM stage						
I/II	25	28	2 (8)	3 (12)	6 (24)	14 (56)
III/IV	32	36	1 (3)	2 (6)	11 (34)	18 (56)
Not evaluable	33	37	0	2 (6)	15 (45)	16 (48)
Lauren classification						
Intestinal	51	57	1 (2)	4 (8)	22 (43)	24 (47)
Diffuse	32	36	2 (6)	1 (3)	7 (22)	22 (69)
Mixed	7	8	0	2 (29)	3 (43)	2 (29)
HER2						
Negative	74	82	3 (4)	7 (9)	22 (30)	42 (57)
Positive	13	14	0	0	8 (62)	5 (38)
Not evaluable*	3	3	0	0	2 (67)	1 (33)

***Abbreviations***: EBV, Epstein-Barr virus; MSI, microsatellite instability; sCIN, surrogate chromosomal instability; sGS, surrogate genomic stability; t-GC, gastric cancer after treatment for Hodgkin lymphoma or testicular cancer; IQR, interquartile range.

* HER2 immunohistochemistry 2+, in situ hybridization not evaluable.

### Classification of molecular subtypes in gastric cancer after treatment for Hodgkin lymphoma or testicular cancer

The molecular subtype distribution of t-GCs is described in Table 2. EBV was positive in 3/90 (3%) t-GCs. The MSI subtype (n = 7, 8%) consisted of six MSI t-GCs and one additional microsatellite stable t-GC with MMR deficiency. This latter tumor showed partly clonal loss of MLH1 and PMS2 staining and partly retained MMR staining in tumor biopsies (Table 3). Of the six MSI t-GCs, three showed a complete loss of MLH1 and PMS2 staining that was explained by *MLH1* promoter methylation. The fourth tumor had a complete loss of MLH1 and PMS2 staining and *MLH1* promoter methylation was absent. A homozygous deletion in chromosome 3, comprising the *MLH1* gene, was detected. This deletion was absent in normal tissue. The fifth MSI tumor showed proficient staining of all four MMR proteins and did not have a methylated *MLH1* promoter. In this tumor, a double somatic mutation in *MSH2* was detected. Germline DNA or normal tissue DNA was not available for this patient, but the levels of mutation detection indicated that these mutations were somatic: both variants were detected at a rate of 20% in a tumor with 40% neoplastic cells. The sixth tumor showed loss of MSH2 and MSH6 staining, but no further analyses could be performed due to insufficient DNA. A total of 32/90 (36%) t-GCs had strong nuclear p53 staining and were classified as sCIN, leaving 48/90 (53%) to be classified as sGS.

**Table 3:** Etiology of mismatch repair dysfunction in gastric cancer after treatment for Hodgkin lymphoma.

MSI t-GC case	MMR loss of staining	MSI	*MLH1* promoter	Mutation analysis
1	MLH1± PMS2± *	MSS	NE	
2	MLH1- PMS2-	MSI	Methylated	
3	MLH1- PMS2-	MSI	Methylated	
4	MLH1- PMS2-	MSI	Methylated	
5	MLH1- PMS2-	MSI	Not methylated	Homozygous deletion including *MLH1*
6	No loss, MMR+	MSI	Not methylated	Double somatic *MSH2* mutation†
7	MSH2- MSH6-	MSI	NE	NE

***Abbreviations*:** MMR, mismatch repair; MSI, microsatellite instability; MSS, microsatellite stability; NE, not evaluable.

* heterogeneous staining: partly clonal loss of staining, partly retained staining.

† Prediction of pathogenicity of MMR gene variant (benign, likely benign, uncertain, likely pathogenic or definitely pathogenic): *MSH2*(c.1511-1G>A), splice site mutation, definitely pathogenic; *MSH2*(c.2251G>A; p.G751R), definitely pathogenic. Both variants were detected at a rate of 20% in a tumor with 40% neoplastic cells.

### Associations of molecular subtypes with characteristics of gastric cancer

The age at t-GC diagnosis differed significantly between subtypes, with median ages for EBV, MSI, sCIN and sGS subtypes of 43, 71, 65 and 52 years, respectively (*p*<0.001). When evaluating the frequency of molecular subtypes according to periods of t-GC diagnosis (Table 2), the frequency of the sGS subtype was 68% in the period 1990–1999, 54% in 2000–2009 and 44% in 2010–2015 (*p =* 0.27).

The frequency of sCIN in gastroesophageal junction t-GCs was 44% compared with 35% in the fundus and corpus, and 25% in antrum (*p =* 0.29, Fig 1). In contrast, the sGS subtype was particularly frequent in the antrum (61%), compared with 49% in the gastroesophageal junction and 47% in the fundus and corpus (*p =* 0.55). Intestinal type carcinomas were 2% EBV, 8% MSI, 43% sCIN and 47% sGS and diffuse type carcinomas were 6% EBV, 3% MSI, 22% sCIN and 69% sGS (*p =* 0.09).

### Associations of gastric cancer molecular subtypes with characteristics of Hodgkin lymphoma or testicular cancer

In Hodgkin lymphoma survivors (n = 66), the subtype distribution was 3% EBV, 11% MSI, 32% sCIN and 55% sGS. (Table 4) In testicular cancer survivors (n = 24), no tumors of the MSI sybtype were detected and the subtype distribution was 4% EBV, 46% sCIN and 50% sGS. We additionally evaluated period of diagnosis of Hodgkin lymphoma or testicular cancer and the association with t-GC subtypes. When Hodgkin lymphoma or testicular cancer was diagnosed before 1990, t-GC was more frequently sGS (63%) than when Hodgkin lymphoma or testicular cancer was diagnosed after 1990 (38%, *p =* 0.03). Only 4% of t-GCs were of the MSI subtype in Hodgkin lymphoma or testicular cancer patients diagnosed before 1990, compared with 15% in Hodgkin lymphoma or testicular cancer patients diagnosed after 1990 (*p =* 0.10).

**Table 4:** Association of molecular subtype distribution of gastric cancer with prior treatment for Hodgkin lymphoma or testicular cancer.

Hodgkin lymphoma / testicular cancer characteristic	Total cohort		EBV	MSI	CIN	GS
	*n* (%)		*n* (%)	*n* (%)	*n* (%)	*n* (%)
t-GC subtype	90	100	3 (3)	7 (8)	32 (36)	48 (53)
Primary cancer						
Hodgkin lymphoma	66	73	2 (3)	7 (11)	21 (32)	36 (55)
Testicular cancer	24	27	1 (4)	0	11 (46)	12 (50)
Year of diagnosis						
1967–1979	19	21	1 (5)	0	6 (32)	12 (63)
1980–1989	37	41	1 (3)	2 (5)	11 (30)	23 (62)
1990–1999	23	26	1 (4)	3 (13)	10 (44)	9 (39)
2000–2009	11	12	0	2 (18)	5 (46)	4 (36)
Abdominal RT						
No	19	21	0	1 (5)	9 (47)	9 (47)
Yes	37	41	0	3 (8)	8 (22)	26 (70)
Unknown	34	38	3 (9)	4 (12)	15 (44)	13 (38)
Alkylating agents						
No	30	33	1 (3)	3 (10)	8 (27)	18 (60)
procarbazine/dacarbazine	23	26	0	1 (4)	9 (39)	13 (57)
Procarbazine/dacarbazine	37	41	2 (5)	3 (8)	15 (41)	17 (46)
Unknown						
Primary treatment category						
Abdominal RT—/ AA -	9	10	0	0	3 (33)	6 (67)
Abdominal RT + / AA -	25	28	0	3 (12)	5 (20)	17 (68)
Abdominal RT—/ AA +	11	12	0	1 (9)	6 (55)	4 (36)
Abdominal RT + / AA +	12	13	0	0	3 (25)	9 (75)
Unknown	33	37	3 (9)	3 (9)	15 (45)	12 (36)

***Abbreviations***: EBV, Epstein-Barr virus; MSI, microsatellite instability; CIN, chromosomal instability; GS, genomic stability; t-GC, gastric cancer after treatment for Hodgkin lymphoma or testicular cancer; IQR, interquartile range; RT, radiotherapy; AA, alkylating agents (procarbazine/dacarbazine).

In patients treated with abdominal radiotherapy, 70% of t-GCs were sGS, in contrast with 47% when no abdominal radiotherapy was given (*p =* 0.15). In t-GCs of patients treated with or without procarbazine/dacarbazine, no differences were seen in sGS frequency (57% vs. 60%, *p =* 0.80). In patients who received both abdominal radiotherapy and procarbazine/dacarbazine, 9/12 (75%) of t-GCs were of the sGS subtype.

### Comparison of molecular subtypes in gastric cancer after treatment for Hodgkin lymphoma or testicular cancer with gastric cancer in the general population

The distribution of subtypes observed in t-GC were compared with published data on the distribution of subtypes in the general population (p-GC) that used the same methodology [27]. Baseline characteristics, including gender, TNM stage, tumor location and Lauren classification were comparable between t-GC and p-GC patients (S1 Table). Mean age at diagnosis, however, was 67 years in p-GC patients and 58 years in t-GC patients.

**Fig 2:**
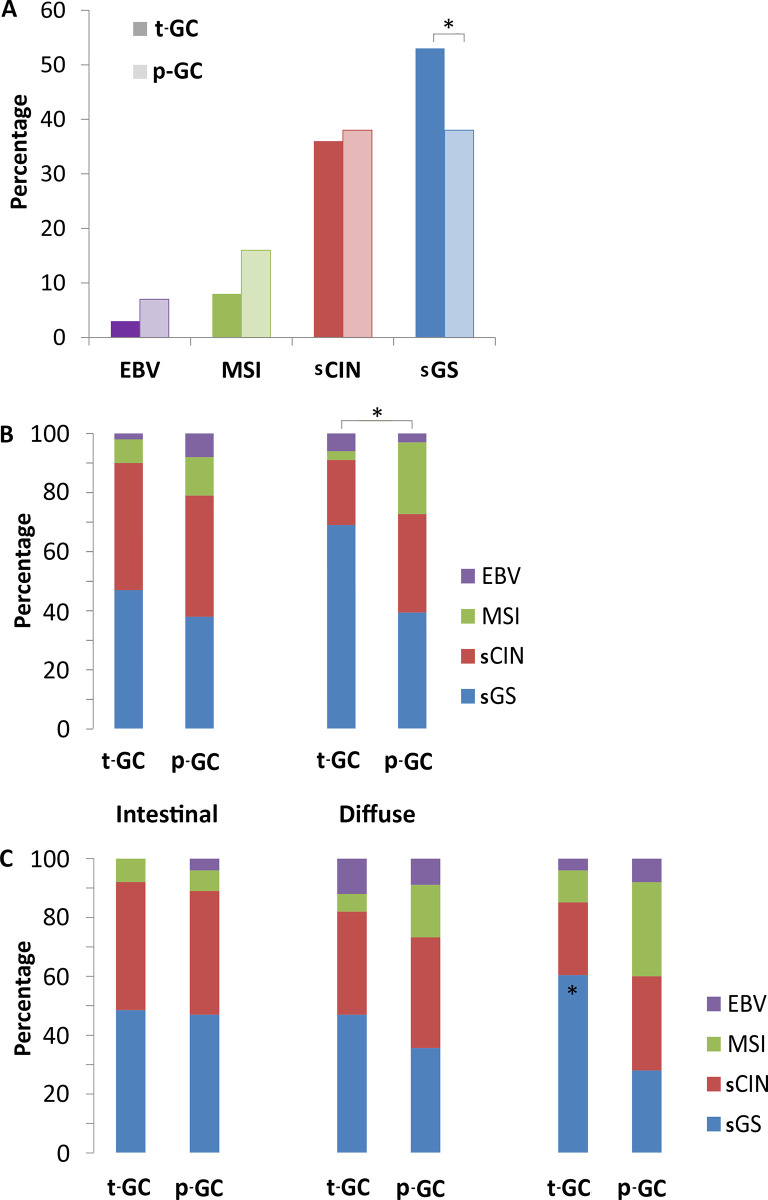
Molecular subtype distribution of gastric cancer after treatment for Hodgkin lymphoma or testicular cancer and gastric cancer in the general population. **A:** overall comparison of molecular subgroup distributions between t-GC and p-GC. **B:** molecular subgroup comparison between t-GC and p-GC after selection of Lauren classification. **C:** molecular subgroup comparison between t-GC and p-GC after selection of gastric cancer location. ***Abbreviations***: EBV, Epstein-Barr virus; MSI, microsatellite instability; sCIN, surrogate chromosomal instability; sGS, surrogate genomic stability; t-GC, gastric cancer after treatment for Hodgkin lymphoma or testicular cancer; p-GC, gastric cancer in the general population; GEJ, gastroesophageal junction. * *p*<0.05, statistically significant difference between t-GC and p-GC groups.

Overall subtype distribution was not significantly different between groups (*p =* 0.10, Fig 2). EBV was present in 3% of t-GCs and 7% p-GCs (*p =* 0.34) and MSI in 8% of t-GCs and 16% of p-GCs (*p =* 0.07). A total of 36% t-GC and 38% p-GCs were sCIN (*p =* 0.68). The only subtype with a significantly different distribution was sGS, which occurred more frequently in t-GCs (53%) than in p-GCs (38%, *p =* 0.04).

When evaluating subtype distribution among intestinal and diffuse types separately, no differences were present between t-GC and p-GC in intestinal type carcinomas (*p =* 0.35, Fig 2). In diffuse type carcinomas, however, the overall distribution in subtypes was different between t-GCs and p-GCs (*p =* 0.02). Specifically in diffuse type t-GCs, the MSI subtype was less frequent (3% vs. 24% in p-GC, *p =* 0.03), sGS was more frequent (69% vs. 39% in p-GC, *p =* 0.03), whereas EBV and sCIN frequencies were not different between groups.

Compared with p-GC, t-GCs located in the antrum were more frequently sGS (61% in t-GC vs. 28% in p-GC, *p =* 0.02), whereas EBV, MSI and sCIN frequencies were not different (EBV 4% vs. 8%, MSI 11% vs. 32%, sCIN 25% vs. 32%, overall test *p =* 0.08). The subtype distribution of gastroesophageal junction and fundus or corpus t-GCs was not different from p-GCs (Fig 2).

## Discussion

This is the first study that describes histopathologic and molecular characteristics of t-GC diagnosed in survivors of Hodgkin lymphoma and testicular cancer. This study was performed primarily to provide initial descriptive data to support future hypothesis-driven research. The main difference between t-GC and gastric adenocarcinoma in the general population is the higher frequency of the sGS subtype, especially within the subgroup of patients who had been treated for their Hodgkin lymphoma or testicular cancer before 1990. In this time period, treatments for Hodgkin lymphoma and testicular cancer were more intensive than in recent years, including higher doses and larger fields of radiotherapy (e.g. extensive abdominal fields) and higher cumulative doses of chemotherapy. Also, t-GCs located in the antrum, an area that receives significant irradiation doses, were more frequently sGS. This suggests an association of sGS adenocarcinoma with prior anticancer treatment. Due to a high degree of missing treatment data, we were not able to demonstrate clear associations of specific treatments with molecular subtypes of t-GC.

An increased frequency of MSI was not observed in t-GC compared with p-GC, unlike what has been observed in therapy-related colorectal cancer [21]. Still, within the small group of MSI t-GCs, double somatic MMR gene mutations could be detected in 2/7 tumors. As a high frequency of double somatic MMR gene mutations was detected in therapy-related colorectal cancer, this may reflect therapy-related carcinogenesis [21]. It can be mentioned that the somatic MMR gene mutations found in the two t-GCs were located outside so called coding poly-A tracts, which was reported as the primary location of somatic MMR gene mutations in sporadic gastric cancer [36]. The poly-A tracts, which are microsatellites that are also present within MMR genes, are highly sensitive to defective MMR. Mutations within these coding poly-A tracts are thought to represent a secondary change due to some other primary MMR dysfunction, such as *MLH1* promoter methylation.

In one of the t-GCs of the MSI subtype, MMR deficiency was only detected in a part of the tumor biopsies. This could be a late event and not a driver event in carcinogenesis. However, only biopsy tissue was available which limited our assessment of the total contribution of MMR deficiency in this case. Therefore we decided to include this tumor in the MSI subtype. The etiology of MMR dysfunction could not be evaluated in this tumor due to limited tissue.

To our knowledge, the association of radiotherapy or alkylating agents with genomically stable gastric cancer, or diffuse type gastric cancer has not been evaluated previously. Several mouse models use alkylating agents as gastric carcinogens, frequently leading to well differentiated intestinal type tumors [18]. However, mouse models of diffuse type cancer also use alkylating agents as carcinogen [37]. In addition, irradiation has been associated with gastric carcinogenesis in mouse models [19]. In humans, the association of abdominal radiotherapy and gastric cancer risk is strong [2, 3, 9–11]. A supramultiplicative interaction of abdominal radiotherapy and alkylating agents with gastric cancer risk has been suggested [2]. Based on these studies, we are not able to assign either radiotherapy or alkylating agents as the major component to induce t-GC.

In our study, evaluations of separate or interactive effects of radiotherapy and/or alkylating agents on t-GC characteristics are limited by missing treatment data of Hodgkin lymphoma and testicular cancer patients. It can be hypothesized that radiotherapy is mainly associated with sGS t-GC, and alkylating agents to a lesser extent. To explain, sGS t-GC was present in a comparable frequency in testicular cancer and Hodgkin lymphoma survivors, and the increased gastric cancer risk in testicular cancer survivors is associated with radiotherapy only [9, 10, 12, 38]. Also, antral t-GCs were frequently of the sGS subtype and this region receives high radiotherapy doses (>30 Gy) after treatment with common fields for both Hodgkin lymphoma and testicular cancer [2, 10].

The selected general population cohort was chosen for comparison because of the the simplicity of the method of gastric cancer subtype evaluation and the comparable baseline characteristics of patients from a Western country. The comparison with this cohort also has several limitations. Firstly, the age difference between t-GCs and the general population cohort limited comparability. This difference is likely attributable to prior anticancer treatment, as the age of onset of therapy-related cancer is often lower than of sporadic cancer in the general population. However, comparison with a younger general population cohort would have given rise to a selective population that developed gastric cancer early due to genetic or environmental factors. Secondly, time periods of gastric cancer diagnosis may have influenced the results, as incidence rates of gastric cancer have changed over time, coinciding with a temporal increase in the proportion of diffuse type and a decrease in the proportion of intestinal type carcinomas. Although the years of diagnosis of gastric cancer in the general population cohort were not reported, a subset of our t-GCs was probably diagnosed earlier, as they were diagnosed between 1982 and 2015 [39]. Thirdly, the methods in our study and in the comparison cohort result in data that are poorly comparable with other studies, except for EBV and MSI analyses (S2 Table) [23–27]. The use of p53 IHC to distinguish between chromosomal instability and genomic stability is debatable, as *TP53* mutations were present in 71% of chromosomally unstable tumors and in approximately 10% of genomically stable tumors within the TCGA set [23]. However, elevated expression of p53 (reverse phase protein array) was suggestive of *TP53* mutations and associated with the chromosomal instability subtype of TCGA tumors. Based on the strong association with missense *TP53* mutations, the definition of aberrant p53 IHC (e.g. the widespread staining of at least 70% of tumor nuclei) was chosen in the comparison cohort [40].

Another limitation of this study is the absence of data on risk factors for gastric cancer, including genetic factors and lifestyle. Also, as reported relative risks of gastric cancer after Hodgkin lymphoma or testicular cancer range from 3–10, leading to an attributable risk of 67–90% ((relative risk–1)/relative risk), approximately 10–33% of our t-GCs are not attributable to prior anticancer treatment but are indeed sporadic [2–4, 9, 10, 41].

The high frequency of the sGC subtype in t-GCs was not explained in our study. There may be (therapy-related) aberrations in these tumors that are specific for these t-GCs. More extensive molecular analyses should be performed to clarify these differences. This was not feasible in this study due a small sample size and material restrictions. These future studies should evaluate therapy-specific aberrations as potential treatment targets. In addition, the molecular characteristics of t-GC should be taken into account when considering the widely discussed options for gastric cancer surveillance in high-risk populations such as survivors of Hodgkin lymphoma and testicular cancer [42, 43].

In conclusion, t-GCs were more frequently classified as sGS subtype compared with p-GC. The pathogenesis of these t-GCs of the sGS subtype remains unkown. The frequency of sGS is higher in t-GC patients who were treated for Hodgkin lymphoma or testicular cancer treatment in an earlier (<1990) time period, when this treatment was more intensive. In contrast with therapy-related colorectal cancer, the frequency of MSI t-GCs was not increased. However, double somatic MMR gene mutations were detected in two out of seven t-GCs of the MSI subtype. These results suggest that at least a subset of t-GCs may have a different pathogenesis than gastric cancer in the general population.

## Supporting information

S1 Table Baseline characteristics gastric cancer after treatment for Hodgkin lymphoma or testicular cancer and gastric cancer in the general population. (DOCX)

S2 Table Distribution of gastric cancer subtypes in literature according to tumor location and Lauren classification. (DOCX)

S1 Fig CONSORT diagram of gastric cancer patients after treatment for Hodgkin lymphoma or testicular cancer. (DOCX)

## Abbreviations

ACRG: Asian Cancer Research Group
EBER: EBV-encoded small RNAs
EBV: Epstein-Barr virus
FFPE: formalin fixed paraffin embedded
HE: haematoxylin-eosin
HER2: Human epidermal receptor 2
IHC: immunohistochemistry
ISH: in situ hybridization
IQR: interquartile range
LOH: loss of heterozygosity
MLPA: multiplex ligation-dependent probe amplification
MMR: mismatch repair
MSI: microsatellite instability
MSS: microsatellite stability
PALGA: Dutch Nationwide Pathology Database
p-GC: gastric cancer in the general population
sCIN: surrogate chromosomal instability
sGS: surrogate genomic stability
SNP: single nucleotide polymorphisms
TCGA: The Cancer Genome Atlas Research Network
t-GC: gastric adenocarcinoma after treatment for Hodgkin lymphoma or testicular cancer
TMA: tissue microarray
WHO: World Health Organization
