# Supplementary material for: Molecular characterization of gastric adenocarcinoma diagnosed in patients previously treated for Hodgkin lymphoma or testicular cancer
Source: PLoS One. 2022 Jul 25;17(7):e0270591. doi: 10.1371/journal.pone.0270591 (PMC9312836; doi:10.1371/journal.pone.0270591)
Supplement: S1 Table — (DOCX) [file pone.0270591.s001.docx]

**Supplementary table 1. Baseline characteristics gastric cancer after treatment for Hodgkin lymphoma or testicular cancer and gastric cancer in the general population.**

| **Characteristic** | **t-GC**  **N=90 (%)** | **p-GC**  **N=104 (%)** | **P-value** |
| --- | --- | --- | --- |
| **Gender**  Male  Female | 70 (78)  20 (22) | 81 (78)  23 (22) | 0.99 |
| **Age at t-GC diagnosis**  Mean (SD) | 58 (15) | 67 (34-95*) | NE |
| **Tumor location**  GEJ  Fundus-Corpus  Antrum  Unknown | 39 (43)  17 (19)  28 (31)  6 (7) | 45 (43)  34 (33)  25 (24)  0 | 0.12 |
| **TNM stage**  I/II  III/IV  Unknown | 25 (28)  32 (36)  33 (37) | 34 (33)  58 (56)  12 (12) | 0.40 |
| **Lauren classification**  Intestinal  Diffuse  Mixed | 51 (57)  32 (36)  7 (8) | 71 (68)  33 (32)  0 | 0.10‡ |
| **HER2**  Negative  Positive  Not evaluable† | 74 (82)  13 (14)  3 (3) | 84 (81)  20 (19)  0 | 0.43 |

**Abbreviations:** t-GC, gastric cancer after treatment for Hodgkin lymphoma or testicular cancer; p-GC, gastric cancer in the general population; GEJ, gastroesophageal junction; SD, standard deviation; NE, not evaluable.

***** range, as no other statistics were reported.

† HER2 immunohistochemistry 2+, in situ hybridization not evaluable.

‡ Chi square test comparing the distribution of intestinal versus diffuse/mixed type between groups.
