# Supplementary material for: Molecular characterization of gastric adenocarcinoma diagnosed in patients previously treated for Hodgkin lymphoma or testicular cancer
Source: PLoS One. 2022 Jul 25;17(7):e0270591. doi: 10.1371/journal.pone.0270591 (PMC9312836; doi:10.1371/journal.pone.0270591)
Supplement: S2 Table — (DOCX) [file pone.0270591.s002.docx]

**Supplementary table 2. Distribution of gastric cancer subtypes in literature according to tumor location and Lauren classification**

| **Population** | **Methods** | **Overall**  **%** | | **GEJ**  **/cardia**  **%** | **Fundus**  **/corpus**  **%** | **Antrum**  **/pylorus %** | **Lauren intestinal**  **%** | **Lauren diffuse**  **%** |
| --- | --- | --- | --- | --- | --- | --- | --- | --- |
| **TCGA 2014 Nature**  N=295  Mixed origin | **Extensive sequencing (e.g. whole exome, RNA)**  **EBV**  **MSI**  **CIN** (somatic copy number aberrations)  **GS** | | 9  22  50  20 | 7  9  65  19 | 14  22  49  16 | 5  27  43  25 | 8  25  60  8 | 7  9  26  58 |
| **Gonzalez 2016 Hum Pathol** N=104  USA | **Immunohistochemistry**  **EBV**: EBER+  **MSI**: MLH1-  **“CIN”**: p53 strong staining (loss not included)  **“GS”**: unremarkable staining | | 7  16  38  38 | 4  7  42  47 | 9  18  38  36 | 8  32  32  28 | 8  13  41  38 | 3  24  33  39 |
| **Cristescu 2015 Nat Med**  N=300  Korea | **Gene expression**  **EBV:** not evaluated  **MSI:** MLH1 loss of expression  **“CIN”**: MSS/TP53- (*CDKN1A/p21, MDM2 loss*)  **“GS”**: MSS/EMT (CDH1 mRNA negative)  MSS/TP53+ (*CDKN1A/p21, MDM2 intact*) | | x  23  36  31 | x  13  28  59 | x  12  36  52 | x  33  28  39 | x  29  40  31 | x  15  31  54 |
| **Setia 2016**  **Modern Pathol** N=146  USA  No GE junction | **Immunohistochemistry**  **EBV**: EBER +  **MSI**: MLH1/PMS2/MSH2/MSH6 –  **“CIN”**: p53 loss or strong staining  **“GS”**: E-cadherin membranous loss  / no aberrations | | 5  16  51  28 | ND | ND | ND | ND | ND |
| **Ahn 2016 Am J Surg Pathol** N=349  Korea | **Immunohistochemistry**  **EBV**: EBER +  **MSI**: MLH1 –  **“CIN”**: p53 loss or strong staining  **“GS”**: E-cadherin membranous loss  / no aberrations | | 7  7  49  36 | 14  0  50  36 | 11  5  48  36 | 2  10  51  37 | 10  11  53  26 | 5  2  43  50 |

**Abbreviations:** EBV, Epstein-Barr virus; MSI, microsatellite instability; CIN, chromosomal instability; GS, genomic stability; ND, not done; GE junction, gastroesophageal junction.
