# Supplementary material for: Molecular characterization of gastric adenocarcinoma diagnosed in patients previously treated for Hodgkin lymphoma or testicular cancer
Source: PLoS One. 2022 Jul 25;17(7):e0270591. doi: 10.1371/journal.pone.0270591 (PMC9312836; doi:10.1371/journal.pone.0270591)
Supplement: S1 Fig — (DOCX) [file pone.0270591.s003.docx]

**Supplementary figure 1. CONSORT diagram of gastric cancer patients after treatment for Hodgkin lymphoma or testicular cancer.**

**
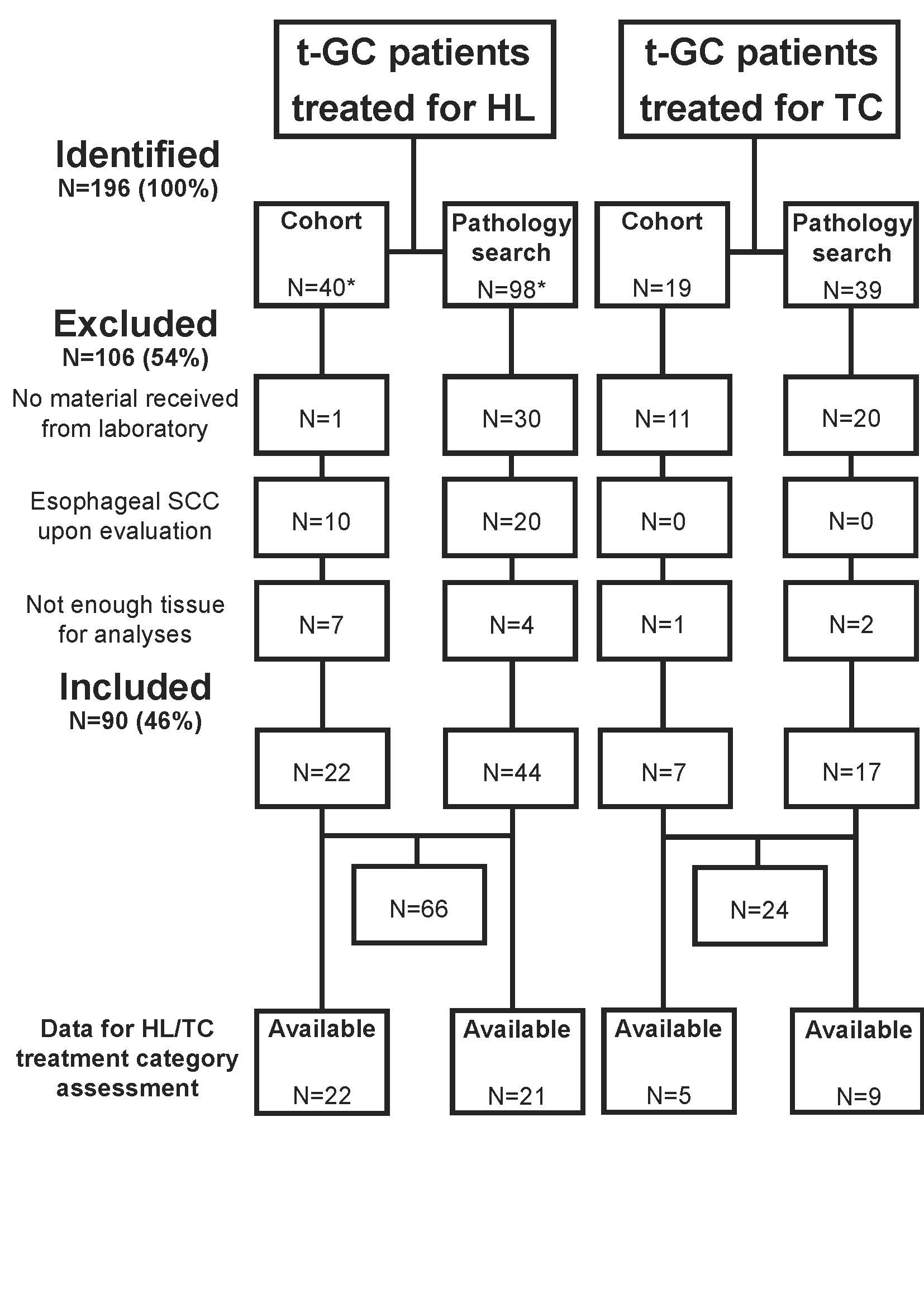
**

***Abbreviations:*** t-GC, gastric cancer after treatment for Hodgkin lymphoma or testicular cancer; HL, Hodgkin lymphoma; TC, testicular cancer; SCC, squamous cell carcinoma.

* These tumors included potential esophageal squamous cell carcinomas, which were excluded from this study after histopathological revision.
